# Emergent categorization in the recognition of black and white paintings through conditional discrimination

**DOI:** 10.1186/s41155-021-00191-y

**Published:** 2021-07-30

**Authors:** Paulo Roberto dos Santos Ferreira, Diana Rasteli Santos, Waldir Monteiro Sampaio, Antonio Carlos Leme Jr., Felipe Maciel dos Santos Souza

**Affiliations:** 1 Universidade Federal da Grande Dourados, Dourados, MS Brazil; 2 Universidade Federal de São Carlos, São Paulo, Brazil

**Keywords:** Categorization, Stimulus equivalence, Paintings, Perceptual classes

## Abstract

**Background:**

The emergent categorization involving paintings by renowned painters and their corresponding names was demonstrated by previous studies. However, the results of these studies suggest that the colors of the pictures may have played a preponderant role, obscuring other aspects of the stimuli that could be more directly related to the style of each painter. To verify this possibility, the present study used the same methodology of Ferreira et al. to investigate the establishment of emergent conditional relations between categories composed of black and white paintings and the names of their authors.

**Method:**

The procedure consisted of the training of relations between each of the ten paintings and an abstract picture, for each of the three painters Botticelli, Monet, and Picasso. Relations between each of the three abstract figures and the printed name of one of the painters were verified in sequence. Finally, tests of relations between five trained and five untrained paintings of each artist and the printed names were conducted.

**Results:**

The participants’ performance suggests that the outcome was properly controlled by aspects pertinent to the paintings that belonged to each painter’s category.

**Conclusions:**

The results reinforced the data obtained previously with colored pictures, suggesting that the process of emergent categorization involving artificial categories of paintings is robust. It also indicates possibilities for future investigations, for example, using stimuli of other artistic productions, such as sculpture and music.

## Introduction

Research into the emergent categorization process can contribute to understanding the interaction of two important basic processes of behavior: the establishment of equivalent classes (Adams, Fields, & Verhave, 1993). Fields, Matneja, Varelas, Belanich, Fitzer, & Shamoun, 2002a; Fields and Garutto, 2009; Mackay, Wilkinson, Farrel, and Serna, 2011) and the categorization or formation of perceptual classes (Avarguès-Weber, Deisig, and Giurfa, 2011; Herrnstein and Loveland, 1964; Berg & Grace, 2011; Watanabe, Wakita, and Sakamoto, 1995; Soto and Wasserman, 2012, Soto and Wasserman, 2014; Watanabe, 2013; Smith and Medin, 1981; Lakoff, 1987; Fields, Reeve, Matneja, Varelas, Belanich, Fitzer, & Shamoun, 2002b). This type of investigation can elucidate new aspects of the behavioral emergent process as well as clarify parameters related to the inclusion of categories in equivalence relations (Zentall, Galizio, & Critchfield, 2002). In addition, studies that investigate the emergent categorization process make it possible to understand aspects that can only be verified in the interaction between the two processes (Ferreira et al., 2018) as well as represent the production of data that are closer to concrete, ecologically valid situations than could produce the investigation of the two processes separately.

Sidman and Tailby (1982) proposed tests to identify conditional discrimination relationships that would be not only unidirectional but evidence of symbolic behavioral emergence, and thus, would be called equivalent relations. These sets of tests are composed of reflexivity, symmetry, and transitivity. For example, consider a person who learns to select an abstract figure A among others available (Dube, 1991) when he is presented with a reproduction of the *Madonna in Glory with Seraphim picture* (among many other paintings). Suppose later that person also learns to select the printed name “Botticelli” among other available printed names of painters (such as “Gauguin” and “Picasso,” for example) in the presence of the abstract picture A. According to the property of reflexivity, that person can select Botticelli when presented with another identical stimulus “Botticelli.” It will also select the *Madonna in Glory with Seraphim* painting when it is presented with the abstract picture A, demonstrating the symmetry property. Finally, it will select the printed name “Botticelli” in the presence of *Madonna in Glory with Seraphim*, which will attest to the property of transitivity. This is the logic present in the formulation of Sidman and Tailby (1982) and has fostered for decades the investigation of symbolic behavior in the area (Sidman, Wynne, Maguire, & Barnes, 1989).

Extending the explanatory potential of Sidman and Tailby (1982), Fields et al. (2002a) taught five adult participants through matching-to-sample tasks to relate elements of different perceptual classes. The perceptual classes were composed of stimuli manipulated along a continuum form of figures of car, truck, and male and female faces. The results of Fields et al. (2002b) suggest that equivalence relations may include elements that share the same perceptual classes with trained stimuli. However, although its results are experimentally quite significant with regard to the defining aspects of the symbolic emergency process, a possible criticism about this line of research lies in the use of stimuli that are not very common in people’s lives, that is, its ecological validity is questionable.

One possibility for advancement in this line of research is the use of culturally relevant stimuli, such as paintings by renowned painters. The experimental question in this line of research is whether the process of categorization (or formation of perceptual classes) would allow individuals to recognize the authorship of unknown paintings based on the learning with other paintings of the same painters. The process of categorization involving paintings by painters, such as Monet and Picasso, has already been obtained in studies with pigeons (Watanabe et al., 1995), mice (Watanabe, 2013), and bees (Wu, Moreno, Tangen and Reinhard, 2013). However, it was only Ferreira et al. (2018) who tested the possibility of symbolic emergence involving the categorization of stimuli paintings by painters in equivalence networks.

In their study, Ferreira et al. (2018) performed a procedure that allowed the establishment of emergent relationships between categories composed by artificial stimuli, paintings by renowned painters, and the names of the corresponding painters—on participants who were unaware of the paintings and their corresponding authorship. The study consisted of two experiments. In experiment I, the relationship between paintings by Gauguin, Botticelli, and Monet and abstract pictures was trained to then train relations between these abstract pictures and the names of the painters. Experiment II consisted of a similar procedure but instead of Gauguin, paintings and the printed name of Picasso were used. In general, the results suggest the establishment of equivalent relations between trained paintings and the names of painters, and satisfactory performance also involving untrained paintings, demonstrating the process of emergent categorization. A relevant result in the study was a greater frequency of hits in Picasso’s paintings. In the discussion of their results, Ferreira et al. (2018) suggested that some structural differences between paintings may have played an important role in this type of difference.

One variable that may have determined part of the results of Ferreira et al. (2018) was the color of the paintings, since it is known that the types of paints and shades characterize the works of certain painters, phases of their artistic production and, often, artistic schools. In this sense, it is relevant to investigate if the process of emergent categorization obtained by Ferreira et al. (2018) could have been determined by the colors of the paintings instead of other characteristics related to the style, such as traces, distributions of themes, and the disposition of the elements in the pictorial representation.

It is interesting to consider that colors do not have a great influence on the categorization of objects (Biederman and Ju, 1988; Davidoff and Ostergaard, 1988; Seamon et al., 1997). In addition, Hanna and Remington (1996) found that color and form discriminations can function independently (see also Stefurak and Boynton, 1986), which may point to the possibility that the process of categorizing paintings may occur without the influence of colors. Conversely, some authors (e.g. Wichmann, Sharpe, and Gegenfurtner, 2002) have found that colors contribute greatly to the recognition memory of natural scenes. However, these same studies have pointed out that colors do not collaborate with the recognition of artificial scenes in the same way that they collaborate with the recognition of natural scenes or objects. In this sense, it is possible that the categorization of frames of realistic tendencies presents a greater dependence on colors than nonrealistic paintings. In any case, the role of colors in the categorization of artificial objects as paintings is a topic that has been insufficiently investigated, especially with regard to their interaction in symbolic processes, such as emergent categorization (Ferreira et al. 2018).

In this way, using black and white paintings in an emergent categorization procedure can elucidate the role that colors played in the results of previous studies. In addition, the use of tests of emergent relations of paintings by painters of the same artistic schools can contribute to the identification of the role of characteristic aspects of certain artistic movements in the production of their paintings. The following questions can be formulated to be answered by the present study: Would it be possible to reproduce the results of Ferreira et al. (2018) using black and white paintings? Would the same kind of variable that would determine the emergent categorization of paintings by a particular painter extend their results to similar style or school painters? Seeking to answer these questions, the relations between potential categories of paintings in black and white and arbitrary stimuli and between these stimuli and the painters’ names were trained. After the training, tests of emergent categorization were carried out involving trained paintings and untrained paintings of the same painters. The results allowed evaluating if colors are necessary to the process of emergent categorization of paintings.

## Method

The experiment reported in this article consisted in the training and test of relations between black and white paintings, abstract pictures, and the names of the painters. Relations between the paintings and the abstract figures were trained, and then were trained between the abstract figures and the names of the painters. Subsequently, untrained relations were tested, including between paintings not shown during the training phase, from the same painters.

### Participants

Ten university students participated in the study, including five women (P1, P5, P7, P8, and P10) and five men (P2, P3, P4, P6, and P9). The mean age for women was 23.6 years, with a standard deviation of 1.14 and the mean age for men was 23.8 years, with an SD of 1.30. The participants did not have any mental disorder, and all had a basic academic repertoire, characteristic of university students, including a good understanding of instructions and skill with the mouse and keyboard. In addition, the lack of knowledge about the paintings and their corresponding authorship was corroborated by the results of all the participants in the first training sessions.

This study belongs to a group of studies that use human subjects and was approved by the university’s research ethics committee (CAAA No. 36513814.0.0000.5162).

### Experimental stimuli

**Fig. 1:**
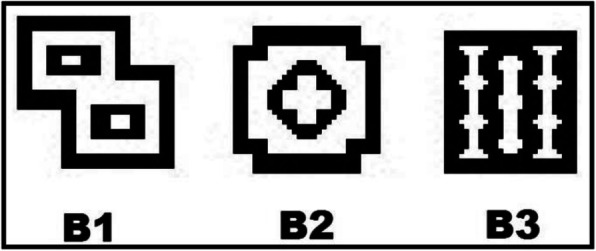
Abstract stimuli experimentally named B1, B2, and B3 (Stromer, McIlvane, Dube and Mackay, 1993)

The stimuli presented in the tasks of conditional discrimination of the experiments were three abstract figures of the set employed by Stromer, McIlvane, Dube, and Mackay (1993) that measured 3 × 3 cm (designated B1, B2, and B3) (Fig. 1), three printed words that referred to painters (C1, C2, and C3) that measured 3 cm tall and 12 cm wide, and 65 black and white paintings (which also contained light-gray and dark-gray regions) that measured 3 × 3 cm. These 45 stimuli were designated among the paintings of the painters as follows: 15 paintings each by the painters Botticelli, Monet, and Picasso (Table 1).

**Table 1:** Paintings used in the experiment

Painting	S	Painting	S
Pablo Picasso			
Head of a Woman (1935)	P1	Nude in Black Armchair (1932)	P9
Reading at a Table (1934)	P2	Three Musicians (1921)	P10
Dora Maar with a Cat (1941)	P3	Girl Before a Mirror (1932)	P11
The Three Dancers (1925)	P4	Jacqueline (1961)	P12
Head of a Woman (1960)	P5	Maya with her Doll (1938)	P13
Jacqueline with Flowers (1954)	P6	The Weeping Woman (1937)	P14
The Dream (1932)	P7	Seated Woman (1927)	P15
The Young Ladies of Avignon (1907)	P8		
Sandro Botticelli			
Madonna in Glory with Seraphim (1469)	T1	Portrait of Giuliano de Medici (1478)	T9
St. Jerome (1498)	T2	Mystic Crucifixion (1497)	T10
Saint Augustine in His Study (1490)	T3	Portrait of a Young Woman (1475)	T11
Virgin and Child with Young St John the Baptist (1515)	T4	Madonna and Child with Two Angels (1444)	T12
Last Communion of St Jerome (1494)	T5	Fortitude (1470)	T13
The return of Judith to Bethulia (1473)	T6	Simonetta Vespucci (1476)	T14
Portrait of a Man with a Medal of Cosimo the Elder (1474)	T7	Portrait of a Young Man (1483)	T15
The Adoration of the Kings (1473)	T8		
Claude Monet			
Women in the Garden (1866)	M1	The Luncheon (1873)	M9
Regatta at Sainte (1867)	M2	Snow at Argenteuil (1875)	M10
Garden at Sainte (1867)	M3	Woman with a Parasol (1875)	M11
La Grenouillère (1869)	M4	The Cliff Walk at Pourville (1882)	M12
The Magpie (1869)	M5	Stormy Sea in Étretat (1883)	M13
Springtime (1872)	M6	The Water Lily Pond (1899)	M14
Boulevard des Capucines (1873)	M7	Bordighera (1884)	M15
Poppies (1873)	M8		

Note: This table presents the stimuli (S) paintings of Pablo Picasso (represented by P), Sandro Botticelli (represented by T), and Claude Monet (represented by M)

### Equipment and experimental environment

To perform the experimental phases, a personal computer with Superlab® software (Abboud and Sugar, 1997) was used to present the conditional discrimination tasks that were programmed by the researchers and to record the participants’ responses through mouse clicks.

Each participant performed the tasks individually. The experiment was applied in a room that was specially designated for this purpose in the university’s experimental psychology laboratory. The participants were seated in padded chairs and responded to stimuli by the computer. The researcher remained in a side room while the participants performed the tasks, waiting to be called by the participant at the end of each session.

### Procedure

**Fig. 2:**
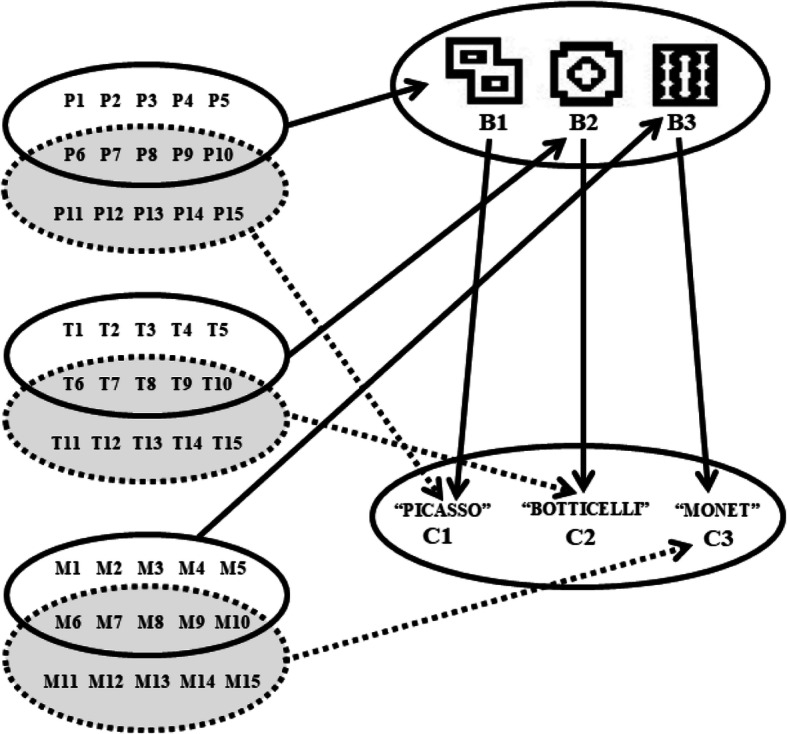
The diagram represents all the trained and tested relations. Ellipses represent sets of stimuli. The continuous arrows indicate training relations and the dashed arrows indicate untrained relations.

The procedure consisted of participants performing tasks on the computer involving training and testing relationships between stimuli in a pre-defined teaching structure (Fig. 2). The responses of the participants consisted of moving the mouse and clicking with the cursor over the stimuli that were presented by the computer. For the beginning of each experimental block, the computer presented instructions programmed by the researcher. The following section presents the description of the tasks that were included in the experimental blocks that composed each phase of the experiment.

### Matching-to-sample tasks

The trials of MTS began with the presentation of a visual stimulus in the center of the upper half of the screen, called stimulus sample. Sample stimuli were paintings or abstract pictures, depending on the type of trial. After the participant responded with the mouse pointer on the sample, three comparison stimuli would appear simultaneously in one of three different positions on the bottom of the screen. The comparison stimuli were an abstract picture or a painter’s printed name, depending on the type of trial. During the training trials, one of these visual stimuli would be designated as the “correct” comparison in terms of its relation to the sample stimulus. The sample and the three comparisons would remain on the screen until the participant clicked on one of the comparisons. The immediate presentation of the comparisons and the fact that the sample remained on the screen characterized this task as a simultaneous matching-to-sample.

Training and test trials composed the experimental phases, depending on what was planned for each phase of the experiment. The participant’s responses were recorded on the computer in all types of trials, tests, and training. In the training trials, if the participant’s choice agreed with the programmed response, a light blue screen would appear with the word “CORRECT” in 72-point Times New Roman font, which was previously defined as a correct response; otherwise, a black screen would appear, which had previously been defined as an incorrect response. After the presentation of the programmed consequence, a new trial was presented. It continued in this manner until the end of the block. Conversely, on the test trials, the computer would record the participants’ responses, but no programmed consequence would follow their choice of comparison. Thus, in the testing blocks, the trials would continue until the end of the session without presenting feedback to the participants.

### Experimental blocks

The experimental blocks were organized according to the following criteria: (1) matching-to-sample trials that were arranged so that the stimuli that were presented for the samples appeared the same number of times; (2) correct comparison did not appear in the same spot on more than 1/3 of the trials; and (3) any given comparison stimulus was randomly distributed across positions on the screen. The Superlab® software allowed systematic randomization of the trials and the position of the comparisons. Thus, participants could not learn the sequence of correct responses based on the comparison stimulus or position on the screen.

The participants were informed whether their responses were correct in the blocks of training trials. When a participant chose a comparison designated as correct, the computer screen indicated accordingly; when the participant chose any of the other comparisons, the computer indicated an incorrect response. In the blocks of test trials, a participant’s response to a comparison stimulus was followed by another trial or by the end of the block. That is, the training trials informed the participants if their responses were correct but the testing trials did not.

The following instructions were shown in 72-point Times New Roman font in capital letters on the computer screen at the beginning of the first block in each phase of the experiments.

Training trial instructions—“Use the mouse to click on the figure shown in the upper half of the screen. Next, three figures (or words) will be shown in the lower half of the screen. Click on one of them. The computer will inform you if you make the right choice. If you are wrong, the black screen will appear. Try to pick the right answer! Good luck! (Click to begin).”

Test trial instructions—“Continue clicking on the figures or words as you did in the previous stage. However, in this stage, the computer will not inform you if your choice was right or wrong. Try to pick the right answer! Good luck! (Click to begin).”

### Experimental phases

The experimental procedure consisted of phases composed of blocks of matching-to-sample trials. The succession of experimental phases occurred according to the pre-established order and criteria.

The first three phases of the procedure were aimed at gradually establishing the required discrimination in phase four and consisted of training blocks that have not necessarily dealt with conditional discrimination contingencies. For example, trials of one of the stimuli for Pablo Picasso as sample (P1, P2, P3, P4, P5, P6, P7, P8, P9, or P10) with the presentation of the abstract stimuli B1, B2 and B3 as comparisons. In this example, the comparison B1 would be the correct comparison when any of the stimuli was presented as a sample. The stimuli that were presented as incorrect comparisons were associated with the other two painters (for example, B2 and B3). The session ended when the following two criteria were achieved: (1) ten trials were presented and (2) the participant chose the correct comparison on six consecutive trials.

The fourth phase included trials from all three previous phases; all 30 paintings were presented as samples and the B1, B2, and B3 stimuli were presented as comparisons. The relations trained were the same as those that had been presented in the previous three phases. Thus, the fourth phase was the junction of phases 1, 2, and 3. The phase ended only after 30 trials have been made and when the participant correctly related each painting by the same painter to the same specifically designated picture on 18 consecutive trials.

Phase 5, which was also a training phase, consisted of trials in which the B1, B2, and B3 stimuli functioned the samples for C1, C2, and C3 comparisons. The session ended when two criteria were achieved: (1) 18 trials were presented and (2) the participant chose the correct comparison on 6 consecutive trials. After phase 5 came the final phase of the procedure.

Phase 6 involved testing the relations between paintings and the names of painters (PC, TC, or MC relations) by using five trained stimuli (TS, trained stimuli) from each painter and adding five exemplars of untrained stimuli (US, untrained stimuli) that were painted by the same artist as a test of recognition and categorization. Thus, the trials used paintings 6–15 (for example, P6 to P15) samples with five stimuli that had been trained (for example, P6 to P10) and five new stimuli (for example, P11 to P15). The comparison stimuli were stimuli C1, C2, and C3, which were designated for each painter according to the experiment. Phase 6 posed 60 equally distributed trials among the shown samples, that is, two trials for each of the 30 presented paintings. The experimental phases are presented in Table 2.

**Table 2:** Phases of experiment

Experimental phase	Criterion	Trained/tested relations
1—Training PB—Picasso	10 trials presented and 6 consecutive correct responses	P1B1, P2B1, P3B1, P4B1, P5B1, P6B1, P7B1, P8B1, P9B1, P10B1
2—Training TB—Botticelli	10 trials presented and 6 consecutive correct responses	T1B2, T2B2, T3B2, T4B2, T5B2, T6B2, T7B2, T8B2, T9B2, T10B2
3—Training MB—Monet	10 trials presented and 6 consecutive correct responses	M1B3, M2B3, M3B3, M4B3, M5B3, M6B3, M7B3, M8B3, M9B3, M10B3
4—Training PB/TB/MB	30 trials presented and 18 consecutive correct responses	P1B1, P2B1, P3B1, P4B1, P5B1, P6B1, P7B1, P8B1, P9B1, P10B1, T1B2, T2B2, T3B2, T4B2, T5B2, T6B2, T7B2, T8B2, T9B2, T10B2, M1B3, M2B3, M3B3, M4B3, M5B3, M6B3, M7B3, M8B3, M9B3, M10B3
5—Training BC	18 trials presented and 6 consecutive correct responses	B1C1, B2C2, B3C3
6—Test PC/TC/MC	No criterion 60 trials were presented (two for each sample stimulus)	P6C1, P7C1, P8C1, P9C1, P10C1, P11C1, P12C1, P13C1, P14C1, P15C1, T6C2, T7C2, T8C2, T9C2, T10C2, T11C2, T12C2, T13C2, T14C2, T15C2, M6C3, M7C3, M8C3, M9C3, M10C3, M11C3, M12C3, M13C3, M14C3, M15C3

Note: The table presents the trained and tested relations: relations between Picasso paintings and stimulus B1 (PB), relations between Botticelli paintings and stimulus B2 (TB), relations between Monet paintings and stimulus B3 (MB), relations between stimuli B and the printed names of painters (BC), relations between Picasso paintings and Picasso printed name (represented by PC), relations between Monet paintings and Monet printed name (represented by MC) and relations between Botticelli paintings and Botticelli printed name (represented by TC)

## Results

### Training

Table 3 shows the number of trials required for participants to achieve the criterion in all training phases performed in the experiment. Participants generally presented a higher proportion of correct responses in the first three training phases. In these phases, it was necessary, on average, that only ten trials for each participant reached the established criterion (six consecutive correct choices). An exception to this result was the number of trials necessary for P7 to reach the criterion, much higher than the average, being 50 for each of the 1-TB and 2-PB phases. However, P7 presented superior performance in phase 3-MB, requiring only 10 trials to reach the criterion.

**Table 3:** Number of training trials in experiment

Participants	1-TB	2-PB	3-MB	4-TB/PB/MB	5-BC
P1	10	10	10	243	33
P2	20	10	10	270	27
P3	20	10	10	120	21
P4	20	10	10	30	21
P5	20	10	10	30	48
P6	20	10	10	154	100
P7	50	50	10	540	21
P8	10	10	10	75	24
P9	10	10	10	360	30
P10	10	10	20	30	15

Note: This table presents the number of trials that were necessary for participants (P1, P2, P3, P4, P5, P6, P7, P8, P9, P10) to meet the learning criterion the relations: relations between Botticelli paintings and stimulus B2 (TB), relations between Picasso paintings and stimulus B1 (PB), relations between Monet paintings and stimulus B3 (MB), and relations between stimuli B and the printed names of painters (BC)

An average of 252 trials was required for six of the 10 participants (P1, P2, P3, P6, P7, P8, and P9) to reach the criterion in phase 4-TB/PB/MB. In contrast, for three participants (P4, P5, and P10), only 30 trials were necessary in this same phase. As a general rule, the number of trials of this phase was always greater than the amount required in the previous three phases. This result was predictable, since the fourth phase was composed of the three previous phases, and, therefore, corresponded to a task with much more complex discriminations.

Table 3 also shows the results for the 5-BC training phase which averaged 34 trials for participants to reach the criterion. More disparate results in this regard were P6 (100 trials) and P10 (15 trials). This result indicates an intermediate performance between the results for phases 1, 2, and 3, and the results for phase 4. In this regard, it is important to consider that this phase comprised the training of the relations between three abstract figures and their corresponding names of the painters, being a task type less complex than that presented in phase 4-TB/PB/MB but more complex than that presented in phases 1, 2, and 3.

### Test

**Fig. 3:**
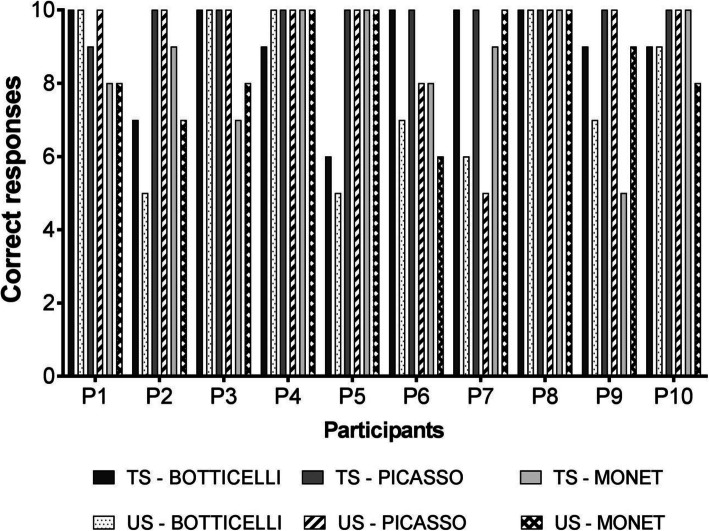
The bars represent the number of correct responses regarding the relations that were performed by the participants on the presented trials in phase 6 that contain the test of relations TC/PC/MC. TS (trained stimuli) 6 to 10 and US (untrained stimuli) 11 to 15 were used as sample stimuli

Figure 3 shows the results for the emerging relationships tested in phase 6. The results suggest that five participants (P1, P3, P4, P8, and P10) presented high performance in transitive relations between trained and untrained paintings and the names of their authors. The number of inconsistent responses to the categories for these five participants was no more than five trials out of the 60 trials presented, with no inconsistent response to P8 and only one incorrect response to P4. The number of incorrect responses for the other five participants was nine, ten, or eleven for the total of 60 trials. Thus, performance was above 81% of correct responses for all participants, which is much higher than chance (33.3%). These results suggest the establishment of emergent categorization.

It is also important to consider that half of the trials presented in phase 6 consisted of relations with US, which characterizes this performance as of emergent categorization. Analyzing separately trials with US from trials with trained stimuli, it is verified that of the 64 incorrect answers presented by all participants, 42 were for trials with US. Even so, a number far above chance, since 300 trials with US were presented, 30 for each participant, that is, were 86% correct and a random distribution would be 33.3%. Still, the proportion of correct responses with trained stimuli was 93%, strongly suggesting the emergence of transitive relationships. In summary, the result suggests that there was emerging categorization involving new black and white pictures of the painters whose pictures were trained.

## Discussion

The study results replicate and extend those presented by Ferreira et al. (2018) and data from both provide strong evidence to support the reality of the emergent categorization. In many ways, it is not surprising to verify this phenomenon, as it represents the combined effect of two behavioral processes widely documented in literature: categorization and stimuli equivalence. It is also important to note the widely known fact that painting recognition is related to the names of its painters, without the need for a direct experience. In addition to all this evidence, an analysis of the complex behavior named as emergent categorization would be promising (see Ferreira et al., 2018). Here, we suggest that the emergent categorization process is not only restricted to specific cases such as colored paintings but also includes black and white paintings.

The results show that the investigation of complex relations such as those established in the formation of equivalence classes can productively benefit from the extensive existing literature that investigated the categorization process (e.g., Medin, & Schaffer, 1978; Medin and Heit, 1999; Wasserman, Kiedinger, & Bhatt, 1988; Soto and Wasserman, 2014; Medin, Lynch, & Solomon, 2000). In addition, the present study demonstrated how the recognition of categories as sophisticated as those composed of sets of black and white paintings can depend on reinforcement contingencies established in the laboratory. These results are quite provocative, since the other studies use categories of stimuli with evident physical similarity (e.g., Fields and Reeve, 2001; Fields, 2016; Herrnstein, Loveland, & Cable, 1976; Herrnstein and de Villiers, 1980; Keller and Schoenfeld, 1950). However, subsequent analysis of the stimuli employed may suggest new approaches to the phenomenon, such as, for example, the role played by the types of representations presented in the paintings (objects, people, etc.) or by types of outlines and other aspects that characterize the painter’s style. Theoretical implications related to this point include a profound reconsideration of the traditional definitions of stimuli (Skinner 1935, 1938/1991).

The participants’ responses suggest the emergent categorizations were established between paintings and their corresponding painters’ names. The participants’ performance suggests that the outcome was properly controlled by aspects pertinent to the paintings that belonged to each painter’s category. It is important to remember that they were never related to any of the paintings. This datum is an extension of Ferreira et al. (2018) who used only colored paintings. The participant’s performance in this study was even more superior compared to what was found in the study by Ferreira et al. (2018). This suggests the importance of replicating the study so as to compare both types of stimuli: colored and black white paintings. This will make it possible to identify whether less color variability of stimuli might have enhanced the learning of aspects pertinent to the involved categories. Following this interpretation of the results, it is important to consider the role played by colors in hindering the establishment of defining aspects of the categories.

As Deng and Sloutsky (2015) pointed out, selective attention processes may play an important role in categorization performance and that would modulate the difference in the performance between colored or black and white stimuli categories. In the same direction, results from Plebanek and Sloutsky (2017) suggest children aged between four and five years can perceive a greater number of characteristics than adults do, for they present “selective attention” (as the authors call it) less frequently. Thus, it would be interesting for future replications of the present study to seek performance differences also between children and adult populations regarding the process of emergent categorization, which involves a more sophisticated process than the isolated process of categorization reported in the studies mentioned.

Lastly, emergent categorization of artistic stimuli in black and white can be considered in its ecological validity when it comes to its applicability in the teaching of three-dimensional stimuli perception. For example, Pinna (2011) observed that the shape of objects precedes colors in the perception process. In this sense, it is possible to consider that to use black and white objects can strengthen the learning of emergent categorization of stimuli, such as shapes. However, reducing the number of characteristics to be identified has an effect yet to be investigated as well as its application in the teaching procedures of symbolic categories composed by artistic objects.

## Data Availability

The data and materials used in the research are available for consultation, which includes software used (Superlab), stimuli, and records of the participants’ responses.

## Abbreviations

M: Monet
MC: Relation between Monet paintings and printed name of the painter
P: Picasso
PC: Relation between Picasso paintings and printed name of the painter
SD: Standard deviation
T: Botticelli
TC: Relation between Botticelli paintings and printed name of the painter
TS: Trained stimuli
US: Untrained stimuli
